# Experience of nurses with using eHealth in Gilgit-Baltistan, Pakistan: a qualitative study in primary and secondary healthcare

**DOI:** 10.1186/1472-6955-12-6

**Published:** 2013-03-02

**Authors:** Saleema Gulzar, Shariq Khoja, Afroz Sajwani

**Affiliations:** 1 The Aga Khan University, Karachi, Pakistan; 2 The Aga Khan University, Nairobi, Kenya

**Keywords:** eHealth, Nurses, Gilgit, Baltistan, Experience, Patient care, Pakistan

## Abstract

**Background:**

To improve the quality of health care in remote parts of Pakistan, a research project was initiated in the mountainous region of Gilgit-Baltistan using information and communication technology to improve patient care and support continuing education of health providers (eHealth). This paper describes the experience of nurses in using eHealth in their routine practices.

**Methods:**

All health centres of Gilgit-Baltistan, Pakistan using eHealth as part of this study, were taken as a single case. These include four primary healthcare centres, three secondary care centres and one medical centre. In-depth interviews were conducted using semi-structured interview guide to study nurses’ perspective about using eHealth, and its perceived impact on their professional lives.

**Results:**

According to the respondents, eHealth enhanced access to care for remote communities, and improved quality of health services by providing opportunities for continuing learning. Nurses also appreciated eHealth for reducing their professional isolation, and providing exposure to new knowledge through teleconsultations and eLearning.

The responses categorized under six major headings include: gaps in health services prior to eHealth; role of eHealth in addressing these gaps; benefits of eHealth; challenges in eHealth implementation; community’s perception about eHealth; and future recommendations.

**Conclusions:**

Low-cost and simple eHealth solutions have shown to benefit nurses, and the communities in the remote mountainous regions of Pakistan.

## Background

Pakistan is a developing country with over 65% of its population living in rural areas [1]. Gilgit-Baltistan region is located in the north eastern part of Pakistan, bordering with Afghanistan, China and India. The area comprises of high mountain peaks, spread over 72,496 square kilometers and population of 1.8 million [2]. The geographic location of Gilgit-Baltistan, and the topographical nature makes it prone to harsh climate like snow storms, wind storms, droughts, floods, landslides and soil erosion [3]. These vulnerabilities, along with the shortage of health facilities, education institutions, and poverty, leads to poor health and quality of life of these communities.

eHealth is defined as the utilization of information and communication technologies (ICT) to support health and health related fields, such as health surveillance, healthcare services, health literature, health education, knowledge and research [4]. Use of ICTs for health is escalating in different parts of the world. In order to assess the efficiency, effectiveness, relevance and affordability of eHealth solutions in remote areas of a developing country like Pakistan, a research project was initiated from January 2010 to June 2011in Gilgit-Baltistan. The project involved health facilities of the Aga Khan Health Service, Pakistan, which is the largest non-government organization in the area.

Literature shows that healthcare providers generally find eHealth beneficial for their professional development, and useful for the patients [5]. It saves their time [6-8], and provides communication and learning opportunities to enhance their knowledge and skills [5,7,9-11]. Use of eHealth also increases communication and interaction between healthcare providers at different levels, reducing professional isolation for providers in remote areas [11,12]. Health providers, especially nurses, also enhance communication and interaction with their patients using eHealth, strengthening the nurse-patient relationship leading towards better care [6,9,13,14]. Healthcare providers also perceive eHealth to cost for the patients, increase their access to healthcare [6,7,15], provide better clinical care and management, and ensure continuity of care [16].

Several papers also report challenges faced by health providers in using eHealth. These challenges include increased workload [10,13,17-20], and dealing with technology-related problems [21,22], such as installation issues [22] and picture and sound quality issues. At times, they have found eHealth costly [7,18] and less user-friendly [6,14]. Healthcare providers have also expressed their lack of confidence and competencies with the use of information and communication technology [6,18,20,23,24]. They have suggested increased provision of trainings and formation of policies regarding the training and education for use of ICT in the curriculum of health education programs [21-24] and also suggested a combination of online and face to face modes to provide healthcare [25]. They have raised concerns about patient’s privacy and security and less direct interaction with online consultations [7,10,14,17].

As part of the project, low cost eHealth solutions were used to connect different levels of health centres in Gilgit-Baltistan for patient management, triage, and referrals. Store-and-forward consultations were carried out using ‘iPath’ software [25] in low-bandwidth setting and live consultations were introduced using ‘ooVoo’ [26] in higher-bandwidth situations. The project was evaluated for the consultation rates, disease and specialty trends, male-female ratio, response time, patient satisfaction, and cost-minimization.

This study presents the perspectives of nurses on the effectiveness of the eHealth intervention. The objective of this study was to explore the experience of nurses in using eHealth at the health facilities in the high mountains of Gilgit-Baltistan, Pakistan.

## Methods

This study reports the qualitative component of a larger Mixed-Methods study conducted to study the impact of eHealth on improving health services in Gilgit-Baltistan. The qualitative component was used to explore the nurses’ perspective about eHealth, and its impact on their professional lives. Purposive sample of nine nurses, who used eHealth regularly in their health facilities, was selected for their current experience with eHealth use, and suggestions for future improvement. A total of nine participants including 4 Community Health Nurses (CHNs); 2 Lady Health Visitors (LHVs); 2 midwives; and 1 field Officer between the ages of 30 to 45 years old, and with 5 to 23 years of experience were interviewed using a semi-structured interview guide. Questions included perceptions of the significance of eHealth in nursing, the possible impact on healthcare system, changes in the role of nurses, facilitators and barriers for successful implementation, and recommendations for strengthening the role of nurses using eHealth. Participants were interviewed on the telephone for approximately 45 minutes. Notes were taken to augment the recording. All interviews were translated from Urdu to English.

Qualitative content analysis was done using NVIVO software, to organize the data findings [27]. The data was analyzed for visible and latent content. Latent content was discovered through the interpretation of underlying meaning of the phrases [27]. The first step of the process required thorough reading of the transcripts several times by the primary researcher and two co-researchers. This facilitated comprehensive understanding of the views of participants and identifying broad categories for arranging the information. Statements from participants’ interviews were coded using NVIVO, and the codes were organized by the researchers to allow major themes to emerge [27]. Rigor was achieved by keeping comprehensive field notes during the data collection and analysis stages, by audio-tapping the interviews and transcribing them verbatim, and by employing investigator triangulation where three investigators collaborated on data collection and analytical decisions. Consensus of all investigators was taken on the latent content and on sections where meaning was not clear. Thematic content was validated by researchers themselves. Ethics approval was obtained from Aga Khan University Ethical Review Committee. and a written consent for participation in the study was signed by each participant.

## Results

**Figure 1:**
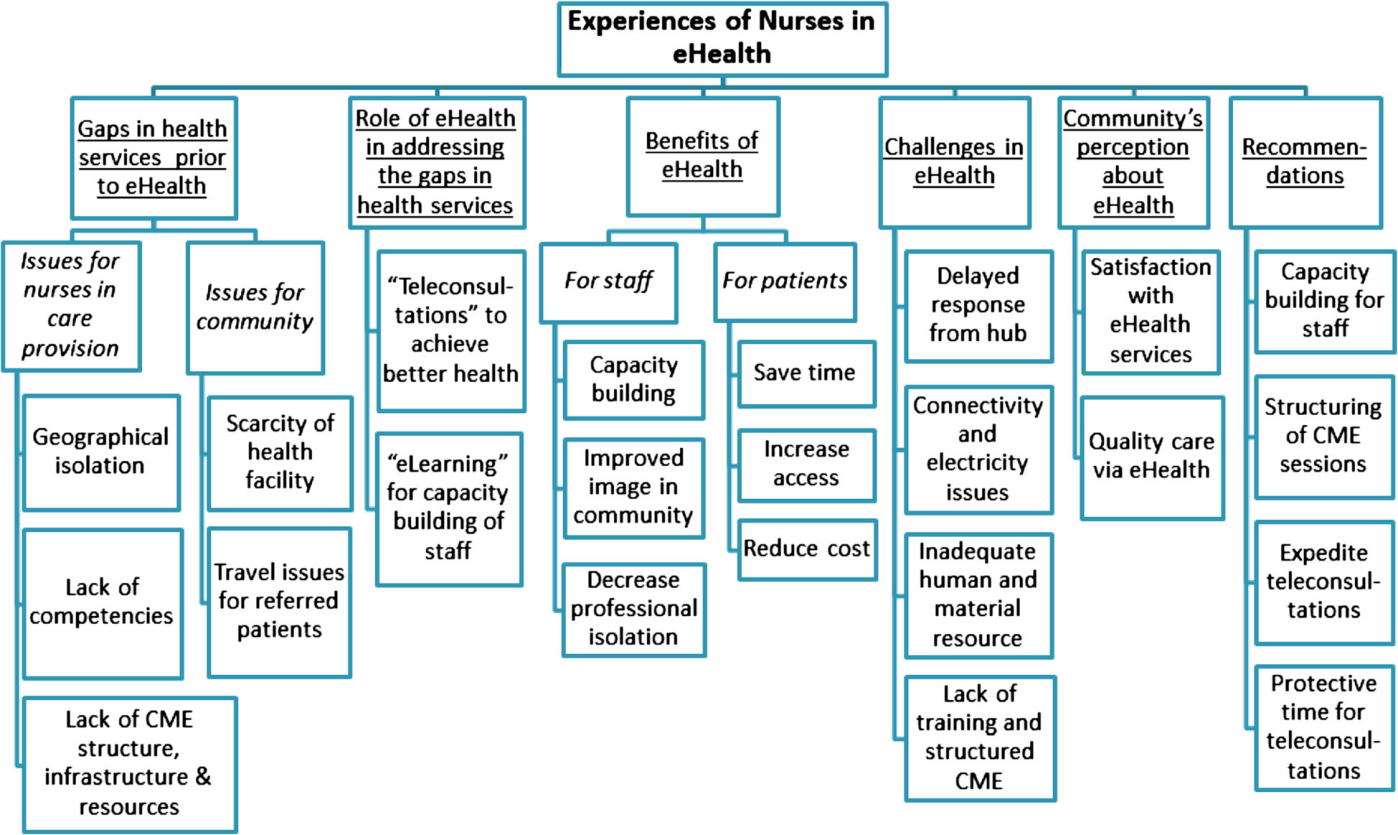
**Themes and sub themes regarding experiences of nurses in eHealth.** Themes that emerged from data collected from Nurses in Gilgit-Baltistan.

Six themes along with their corresponding sub-themes emerged from the data analysis capturing the essence of the experience of nurses using eHealth in their respective health facilities during January 2010 to June 2011 (see Figure 1).

The detail of each theme is explained below:

1. Gaps in health services prior to eHealth

Nurses talked about the issues they encountered in providing healthcare, and the problems faced by the community in acquiring these services.

1.1. Issues for nurses in care provision

Nurses shared their lack of communication with other health care professionals as a barrier in providing quality care by limiting their opportunities for knowledge enhancement. The main reason identified was the distance between health facilities and lack of internet or telephone facilities at the centres. Nurses also highlighted their lack of competencies in certain areas which hindered provision of holistic care to the patients at their respective health facilities.

According to one participant, *“Our health centre is located in such an area that due to difficult travel we remain cut off from other healthcare facilities, which caused us problem in sharing patient data and seeking advice accordingly, so we had no other option than referring the patient to next level health care facility.”*

1.2. Issues for community

Nurses shared that due to shortage of healthcare facilities in their region, community found it very difficult to access the services. They had to travel from far-flung areas to reach the next level facility causing delays in care, increasing cost, and difficulties for the sick patients to travel on difficult roads in harsh weather.

According to one participant, *“Because of (our) lack of expertise to handle difficult cases at our health centre, we had to refer pregnant mothers to other centres and it was very difficult for them to travel in such health conditions.”*

2. Role of eHealth in addressing the gaps in health services

eHealth helped nurses consult difficult cases with the specialists for providing better care to the communities. These discussions, along with capacity-building sessions helped enhance their knowledge and skills.

One participant mentioned that, *“Our staff has done approximately 28 (online) consultations with (doctors at) the secondary care units. We have received timely feedback from the secondary care units. Our staff in ‘X’ health centre received a patient with a skin infection. The nurse took a picture from camera of the infected area, fed the details in the laptop and sent the information and the picture for consultation. The patient informed everyone in the community that (the staff) used machines to take my picture and get quick advice from the consultant to treat my problem.”*

3. Benefits of eHealth

3.1 Benefits to staff

Many nurses considered eHealth as an opportunity for regular learning. Because of teleconsultations, eLearning sessions, and internet browsing, they felt better equipped with recent treatment regimen and case management. Image of nursing profession has always been a concern for nurses in Pakistan. Many nurses and LHVs said that eHealth has not only improved their reputation in the community but also resulted in better communication and coordination between healthcare providers and hence reduced their professional isolation.

A senior nurse reported, *“eHealth has improved (nurses’) image in the community; recently we celebrated Nurses Day here, and this information was shared via newsletter. People are accepting us, even when doctors see us using computer, they get surprised. Through the newsletter, they got to know that nurses also use eHealth applications; so things are improving and people now know that nurses can use eHealth for improving health (services).”*

3.2 Benefits to patients

Seeking healthcare used to be cumbersome for people in Gilgit-Baltistan due to climatic and geographical constraints. According to a CHN, *“There are many transportation issues, or the next level facility is at times too far, and at times requires patients to travel across the lake on a boat. So through eHealth we solve this problem and save (patients’) travel time and cost. We also provide better consultation and good care to patients and manage their health problems.”*

4. Challenges in eHealth

Nurses in our study reported some issues which at times were bottle neck to using eHealth, such as technological problems, connectivity issues, lack of electricity, limited number of computers, and shortage of healthcare staff trained in eHealth. At times, delayed response from consultants also caused problems in providing timely care to the patients.

A senior nurse reported, *“We have only one computer which is shared with doctors as well, so (nurses) are hesitant to access the computer. One computer is not sufficient to be able to gain knowledge and enhance our learning. We need more resources. … This is a modern period and until and unless staff gets new information, they won’t be able to provide good care.”*

Many nurses showed desire to update their knowledge through continuing education program, but due to lack of a structured continuing education program, they were not able to attend the sessions regularly.

5. Community’s perception about eHealth

Community found care provision through eHealth gratifying, as they were getting specialized health care at their door steps.

6. Recommendations

According to a participant*, “Patients are extremely satisfied, since their travel time and cost is saved. Without eHealth, patients travelled long distances with other family members to get the treatment. They spent 3–4 hours to travel and approximately 5000 rupees (60 US$) to get the treatment. Now with eHealth they only pay 200 ropees (2.50 US$), and they are happy with this.”*

Participants were interested in learning about and applying eHealth applications in their practice settings. However, they listed some recommendations to enhance the utilization of eHealth in health facilities. These include frequent capacity building for staff, including hands-on trainings and refreshers, need to expedite teleconsultations by reducing response time by the consultant for emergency cases, and regular programs with tertiary teaching hospital for online consultations and learning to maximize the utilization of the available resources. Nurses mentioned about their increased workload following the introduction of eHealth and suggested to get protective time for eHealth related activities.

One CHN shared, *“There should be dedicated persons to respond to consultations at the hub centres… so that we can get timely reply on the cases that we refer. The staff (at the hub centres) gets busy in their patient care and there is no dedicated person who could check and respond to the cases referred by the primary care facilities. It would be helpful if a person takes responsibility for getting quick responses. Sometime when patients come back to us for follow up of their consultation, we have to say sorry to them because we don’t receive timely reply for our desired queries and consultations.”*

## Discussion

Nurses mentioned significant advantages of eHealth for themselves and for the community. Patients got treated and received quality nursing care at their nearest facility. This finding supports existing evidence, which shows improved access to specialized care in remote areas [14]. Looking at the scenario of Gilgit-Baltistan, where people have to travel long distances to the next care facility for specialized care, and are accompanied by their attendants, the cost of travel becomes added burden on them. eHealth removes these inconveniences and additional costs for the patients. Literature also reveals that patients find seeking care through teleconsultation very cost effective [10].

Findings of this study also show that when patients were receiving timely care at their own vicinity, it resulted in building a good rapport between patients and nurses. Nurses are the first point of contact when patients seek care at these health facilities. Other studies have shown loss of direct contact between the patients and health care providers including nurses [6], Nu in the present study, it is important to highlight that nurses were the ones who had direct contact with the patients, and they uploaded the cases after taking necessary history and performing physical examination of patients. In return they get consultant's advice and the diagnosis and treatment. Hence, decrease in face-to-face contact was not a concern in this case. However, literature has suggested that building a relationship of trust in such settings requires different strategies [6,19].

The findings also reveal that eHealth initiative played a great role in the professional development of nurses. Nurses clearly expressed that use of eHealth not only enhanced their knowledge regarding information and communication technology, but also upgraded their health related knowledge as they are in direct contact with the consultant to seek information, in case they need clarifications about medical terminologies and treatment. Literature also supports that ICT facilitated nurses’ work and gave them more knowledge about ill person and improve nursing care [6]. Use of ICT allowed working conditions to be more organized and eventually reduced stress [6]. This provided significant satisfaction to the patients as they received holistic care, and in addition, healthcare providers including nurses felt content with this innovative way to connect with the patients [14,21]. Hence role of the nurse becomes apparent with introduction eHealth. However, other study asserts that the role of nurses does not change in telehealth, only the way in which it delivered and location differs [19].

The present study also uncovered the fact that eHealth reduced the social isolation of nurses by connecting them with experts in the field of health from different geographical areas. eHealth has also helped build strong rapport among the health care providers [21].

As far as the challenges are concerned, technology-related problems were highlighted repeatedly. At the beginning, nurses were not comfortable with the technology. However, relevant eHealth training and on-going and timely facilitation by locally assigned eHealth coordinators, made nurses comfortable to use the eHealth applications. Study findings support that the participants who were unfamiliar with technology are able to use it with training and continuous supervision, similar to what’s seen in other studies [19]. The most important barriers to implementing eHealth were lack of suitable training, cost of equipment, and increase in work load [18]. The huge benefits of eHealth to the community and health providers also call for changes in the staffing and structure of health facilities. This includes allotting dedicated time for telehealth services and placing computers in locations accessible to nurses. These changes should enable the nurses to obtain quick advice about the diagnosis and treatment of their patients, without forcing the staff at the secondary care facilities to leave their routine tasks.

Our study findings also support the evidence that eHealth has the potential of reducing social isolation by facilitating access to learning and educational resources. This study recognized the potential benefits of eHealth in rural health setting, but it also highlights important barriers and challenges to its wider function amongst nurses particularly, such as technical issues and lack of training. It is crucial that while planning eHealth initiatives in primary care, health care providers’ needs should be considered to avoid issues with acceptance of technology, and obtain maximum benefits.

Although the study reveals important information, the data has been collected from nine nurses using eHealth regularly. This information may not be generalizable to many parts of Pakistan and in areas with very different infrastructure, but the learnings from this study can provide guidance in planning of eHealth programs in many similar settings. Moreover, an in-depth interview with a large number of health providers after the study has been implemented broadly, could reveal more generalizable results.

## Conclusions

The introduction of eHealth has opened new and innovative ways to provide quality health care to the community. The satisfaction level of the community also enhanced due to increased access and quality care available at their own vicinity. Moreover, nurses described eHealth as a solution to reduce their professional isolation as they are now exposed to new knowledge through teleconsultation and eLearning. Therefore, it is important that eHealth should be introduced as part of nursing practice and nursing curriculum.

Overall, nurses perceived eHealth experiences useful as well as challenging in terms of providing patients care. On-going training in using eHealth is crucial to augment nurses’ confidence and support their professional development.

## Competing interests

The authors declare that they have no competing interests.

## Authors’ contributions

SK conceived and designed the study and participated in the data analysis. SG performed the data analysis, interpretation of data and interviewing with the nurses. AS was involved in coordinating the study and drafting the manuscript. All authors have read and approved the final manuscript.

## Pre-publication history

The pre-publication history for this paper can be accessed here:

http://www.biomedcentral.com/1472-6955/12/6/prepub
